# A Novel TCGA-Validated, MiRNA-Based Signature for Prediction of Breast Cancer Prognosis and Survival

**DOI:** 10.3389/fcell.2021.717462

**Published:** 2021-09-13

**Authors:** Baoxing Tian, Mengjie Hou, Kun Zhou, Xia Qiu, Yibao Du, Yifan Gu, Xiaoxing Yin, Jie Wang

**Affiliations:** ^1^Department of Breast Surgery, Tongren Hospital, Shanghai Jiao Tong University School of Medicine, Shanghai, China; ^2^Shanghai Key Laboratory of Tissue Engineering, Department of Plastic and Reconstructive Surgery, Shanghai 9th People’s Hospital, Shanghai Jiao Tong University School of Medicine, Shanghai, China; ^3^Department of General Surgery, Jing’an District Center Hospital, Fudan University, Shanghai, China

**Keywords:** breast cancer, miRNA, prognostic model, biomarker, signature, TCGA

## Abstract

Breast cancer (BC) is the most common cancer affecting women and the leading cause of cancer-related deaths worldwide. Compelling evidence indicates that microRNAs (miRNAs) are inextricably involved in the development of cancer. Here, we constructed a novel model, based on miRNA-seq and clinical data downloaded from The Cancer Genome Atlas (TCGA). Data from a total of 962 patients were included in this study, and the relationships among their clinicopathological features, survival, and miRNA-seq expression levels were analyzed. Hsa-miR-186 and hsa-miR-361 were identified as internal reference miRNAs and used to normalize miRNA expression data. A five-miRNA signature, constructed using univariate and multivariate Cox regression, was significantly associated with disease-specific survival (DSS) of patients with BC. Kaplan–Meier (KM) and receiver operating characteristic (ROC) analyses were conducted to confirm the clinical significance of the five-miRNA signature. Finally, a nomogram was constructed based on the five-miRNA signature to evaluate its clinical value. Cox regression analysis revealed that a five-miRNA signature was significantly associated with DSS of patients with BC. KM analysis demonstrated that the signature could efficiently distinguish high- and low-risk patients. Moreover, ROC analysis showed that the five-miRNA signature exhibited high sensitivity and specificity in predicting the prognosis of patients with BC. Patients in the high-risk subgroup who received adjuvant chemotherapy had a significantly lower incidence of mortality than those who did not. A nomogram constructed based on the five-miRNA signature was effective in predicting 5-year DSS. This study presents a novel five-miRNA signature as a reliable prognostic tool to predict DSS and provide theoretical reference significance for individualized clinical decisions for patients with BC.

## Introduction

Globally, breast cancer (BC) is the leading cause of cancer mortality among women. It is estimated that 2.1 million new BC cases were diagnosed worldwide in 2018, accounting for almost one in four cancer cases (Bray et al., 2018). BC is clinically categorized into three types based on tumor hormonal status: luminal-like, human epidermal growth factor receptor 2 (HER2)-positive, and triple-negative BC. Luminal-like and HER2-positive BC are driven by the hormone receptors and HER2, while triple-negative BC does not express receptors for either hormones or human epidermal growth factor. The classification of BC by hormonal status has important therapeutic implications, as luminal-like cases may be treated using hormonal therapy, which is ineffective against HER2-positive and triple-negative breast cancer (Slamon et al., 1987; Fisher et al., 1989; Perou, 2011). Although multiple prognostic tools, including Oncotype Dx, MammaPrint, Theros, MapQuant Dx, and PAM50, have been developed for routine prognostic application in luminal-like BC, none is capable of predicting prognosis in all types of BC (Krop et al., 2017). While BC hormonal receptor status, HER2 status, tumor size, and the extent of lymph node metastases are independent prognostic indicators, these factors alone are inadequate guides for personalized BC treatment.

MicroRNAs (miRNAs) are small non-coding RNA molecules that regulate gene expression (Liu et al., 2014). Multiple studies have shown that miRNA function has a critical role in tumorigenesis, progression, and therapy (Rupaimoole and Slack, 2017). For instance, high expression levels of hsa-miR-21 are associated with specific BC clinical features, including advanced tumor stage, lymph node metastasis, and shortened patient survival (Yan et al., 2008). In addition, hsa-miR-210 and hsa-miR-221 have been linked with BC invasion and poorer prognosis of patients with BC (Volinia et al., 2012). Other miRNAs associated with prognosis in BC include hsa-miR-155, hsa-miR-206, hsa-miR-133b, hsa-miR-10b, let-7b, hsa-miR-30a, and hsa-miR-505 (Chen et al., 2012; Cheng et al., 2012; Ma et al., 2014; Parrella et al., 2014; Quan et al., 2018; Wang et al., 2019; Yao et al., 2019). However, many previous studies have been limited by small numbers of participants or a lack of extensive study scope, which has limited the translation of their findings into clinical application; for instance, the research results of the hsa-miR-30a only apply to triple-negative breast cancer (Cheng et al., 2012), and the study of the hsa-miR-10b has scarce representation in the population of cases classified as low risk by the Nottingham Prognostic Index (Parrella et al., 2014).

Recent research shows that multiple-miRNA signature (model) could predict the prognosis of some cancer; for instance, four-miRNA classifier is a reliable prognostic prediction tool for overall survival (OS) in lymph node-positive locoregional esophageal squamous cell carcinoma (Wen et al., 2021), a six-miRNA-based classifier is a reliable prognostic and predictive tool for disease recurrence in patients with stage II colon cancer (Zhang et al., 2013), and a robust six-miRNA prognostic signature (Zhao and Cui, 2020) and a novel seven-miRNA prognostic model are reliable to predict OS (Lu et al., 2019) for head and neck squamous cell carcinoma. It is worth mentioning that there were three multi-miRNA prognostic models for breast cancer. Lai et al. (2019) had constructed a novel six-microRNA-based model to predict OS, Tang et al. (2019) constructed a 17-miRNA-based model for OS and a 13-miRNA-based model for recurrence-free survival, and Lu et al. (2020) constructed a three-miRNA-based model for OS. However, all of the three multi-miRNA prognostic models for breast cancer lack a comprehensive analysis and do not identify the risk subgroup patients to tailor adjuvant chemotherapy.

The Cancer Genome Atlas (TCGA)^1^ project has applied extensive genomic sequencing and bioinformatics analyses to catalog human cancer-causing mutations in large cohorts, and the resulting datasets are publicly available (Tomczak et al., 2015). In this study, we took advantage of miRNA-seq and clinical datasets from TCGA for use in constructing a novel prognosis prediction model for disease-specific survival (DSS) in patients with BC.

## Materials and Methods

### MiRNA-Seq and Clinical Datasets

Level 3 miRNA-seq data and accompanying clinical datasets were obtained through TCGA Genomic Data Commons portal (GDC, see text footnote 1). Samples from 962 female patients with BC cataloged by TCGA, and for which clinical features and miRNA-seq data were available, were selected for further analyses. The BC dataset from TCGA used was last updated on June 13, 2018. The results reported here are completely based on data generated by TCGA Research Network.

### Expression Data Normalization

Perl Critic software was used to merge miRNA-seq files and the Normalize Quantiles (edgeR, R package) was used to normalize miRNA expression levels. A total of 1,601 miRNAs were filtered out. Reference miRNA candidates were selected if they met all of the following inclusion criteria (Zhan et al., 2014):

(1) Mean (normal) > 100 and mean (tumor) > 100
(2) $\frac{Standarddeviation\left(normal\right)}{Mean\left(normal\right)}<0.5$ and $\frac{Standarddeviation\left(tumor\right)}{Mean\left(tumor\right)}<0.5$
(3) $\frac{Mean\left(tumor\right)}{Mean\left(normal\right)}<1.3$ and $\frac{Mean\left(normal\right)}{Mean\left(tumor\right)}<1.3$

Ultimately, hsa-miR-186 and hsa-miR-361 were chosen as internal reference molecules (Table 1) and used to normalize miRNA expression levels, based on the following formula:


$$\begin{array}{c} Expressionlevel\\ \;=log\left(\frac{\text{hsa}-miR-XXX}{\frac{1}{2}\left(\text{hsa}-\text{miR}-361+hsa-miR-186\right)}+1,2\right) \end{array}$$

**TABLE 1:** Selection of internal reference miRNA.

Variables	Mean		SD		SD/M		M (N)/M (T)	M (T)/M (N)
	Normal	Tumor	Normal	Tumor	Normal	Tumor		
Hsa-miR-361	970.445	878.666	362.230	370.779	0.373	0.422	1.104	0.905
Hsa-miR-186	949.227	866.105	349.374	388.574	0.368	0.449	1.096	0.912

*SD, standard deviation; M, mean; N, normal; T, tumor.*

The logarithm of the data is convenient for statistical analysis (Olivier et al., 2008; Sorrentino, 2010), which can reduce the absolute value of the data and facilitate calculation. Logarithm will not change the nature and correlation of the data but only compress the scale of the variables. Seventy-five differentially expressed miRNAs, including 14 up-regulated and 61 down-regulated, were screened out using edgeR (log_2_|fold change| > 1, adjusted *P*-value < 0.05).

### Risk Prognostic Model Construction and Evaluation

The dataset (*N* = 962) was used to develop a clinical prediction model, and half of the dataset (*N* = 481) were randomly selected as a validation dataset, which was used for verification of the accuracy of the miRNA-based prognostic model as a predictor of DSS of patients with BC. Univariate and multivariate Cox proportional hazard models were constructed as risk prognostic models and area under the curve (AUC) analysis conducted to evaluate their accuracy.

### Statistical Analyses

Statistical analyses were conducted using SPSS statistical suite version 25.0, ActivePerl version 5.26.3, and R version 3.6.1. Statistical significance was defined as a two-sided *P*-value or adjusted *P*-value < 0.05, except in univariate Cox proportional hazard analysis, where *P* < 0.1 was considered significant. The main outcome in this study was DSS, and the event time of DSS was defined according to the guidelines for time-to-event end point definitions in BC trials (Gourgou-Bourgade et al., 2015). The DSS event times for the individual patients enrolled in this retrospective study were manually retrieved from TCGA clinical records and a previous study (Thorsson et al., 2018). MiRNAs with prognostic value identified using univariate Cox proportional hazards models (*P* < 0.1) were further analyzed by multivariate Cox regression (default settings: backward, conditional, and entry 0.05; removal 0.10), and miRNAs identified by this analysis were used to construct a formula for calculation of prognostic risk scores. The results of univariate and multivariate Cox regression are presented as hazard ratio (HR) with 95% confidence interval (CI). Receiver operating characteristic (ROC) curve and distance on curve [DOC, equaling the square root of (1 − Sensitivity)^2^ + (1 − Specificity)^2^] were performed to calculate cut-off points. Next, BC cases were categorized as “low risk” or “high risk” based on risk score higher or lower than the cut-off point. Chi-square analysis was used to assess the correlation between BC clinicopathological features and risk subgroups. Kaplan–Meier (KM) analyses were applied to generate survival curves, and log rank test was used to establish the significance of differences between curves. A prognostic nomogram to predict individual survival based on an miRNA signature and clinical risk factors was constructed by Cox regression. The accuracy of the risk prognostic model was tested using AUC (95% CI) values.

## Results

### Identification of MiRNAs Associated With DSS in Patients With BC

**FIGURE 1:**
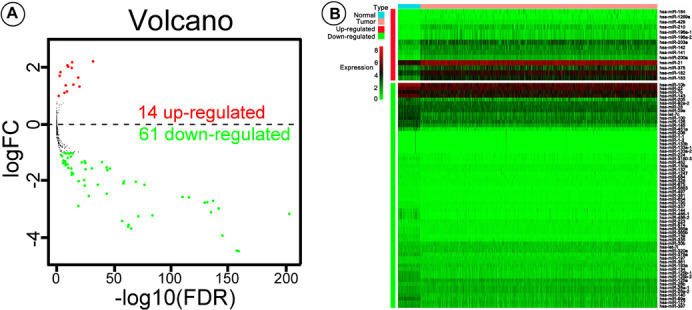
Volcano plot and pheatmap showing differentially expressed microRNAs (miRNAs). **(A)** Values plotted are mean normalized signal values (log10-scaled) for control (*x*-axis) and experimental (*y*-axis) groups. Red and green points correspond to twofold-change up/down, respectively (*P* < 0.05). **(B)** The pheatmap R package was used to plot expression levels of the differentially expressed miRNAs in breast cancer (BC) and adjacent tissue; red and green indicate that miRNA was expressed at high or low levels, respectively.

A total of 1,601 miRNAs were filtered out, and 75 miRNAs were detected as differentially expressed using the edgeR package, including 14 up- and 61 down-regulated miRNAs (Figure 1A), and the expression levels of the 75 miRNAs were shown using the pheatmap package (Figure 1B). Further, univariate Cox proportional hazard analysis identified 23 miRNAs with prognostic value, five of which (hsa-miR-574, hsa-miR-30b, hsa-miR-224, hsa-miR-210, and hsa-miR-130a) were determined by multivariate Cox regression to be the optimum prognostic model for predicting DSS risk in patients with BC (Table 2). Risk scores were calculated using the formula:

**TABLE 2:** Variables in the equation by multivariate Cox regression.

Variables	Coefficient	SE	*P*-value	HR	95.0% CI for HR	
					Lower	Upper
Hsa-miR-574	2.682	0.902	0.003	14.618	2.496	85.624
Hsa-miR-30b	–0.904	0.446	0.043	0.405	0.169	0.970
Hsa-miR-224	1.102	0.490	0.024	3.010	1.153	7.858
Hsa-miR-210	0.525	0.201	0.009	1.691	1.140	2.508
Hsa-miR-130a	–4.738	1.499	0.002	0.009	0.000	0.165

*SE, standard error; HR, hazard ratio; CI, confidence interval.*

**FIGURE 2:**
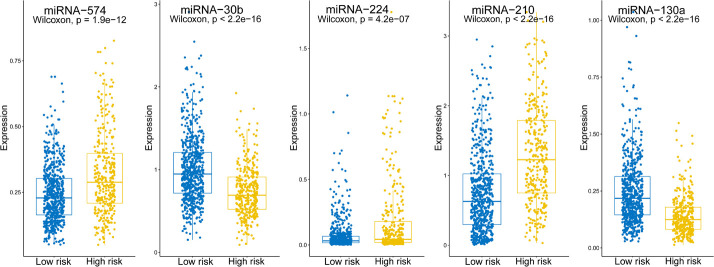
Expression levels of the five screened miRNAs. Boxplots show the expression levels of the five miRNAs in the high- and low-risk groups.

2.682 × hsa-miR-574 − 0.904 × hsa-miR-30b + 1.102 × hsa-miR-224 + 0.525 × hsa-miR-210 − 4.738 × hsa-miR-130a indicating that hsa-miRNA-574, hsa-miRNA-224, and hsa-miRNA-210 were associated with higher risk scores, whereas hsa-miRNA-30b and hsa-miRNA-130a were linked with lower risk scores. Expression levels of the five miRNAs are shown separately in Figure 2. The risk score, −0.197, was calculated as the cut-off point *via* ROC curve and DOC (DOC = 0.436) (Table 3). ROC curves for various risk score cut-off points are shown in Table 3. Using this model, patients were grouped into high-risk (*N* = 338) and low-risk (*N* = 624) groups, according to the cut-off point.

**TABLE 3:** ROC curve for various cut-off levels of the risk score.

The risk score	Sensitivity (95% CI)	Specificity (95% CI)	DOC
−0.230401325	0.740 (0.705–0.775)	0.649 (0.614–0.684)	0.437
−0.197567209	0.720 (0.695–0.755)	0.665 (0.630–0.700)	0.437
−0.196784704	0.720 (0.695–0.755)	0.666 (0.631–0.701)	0.436 (cut-off)
−0.174387142	0.700 (0.665–0.735)	0.683 (0.648–0.718)	0.437
−0.133806071	0.680 (0.645–0.715)	0.703 (0.668–0.738)	0.437

*AUC: 0.714 (95% CI: 0.645–0.783), P = 0.000. DOC, distance on curve equaling square root of (1 − Sen)^2^ + (1 − Spe)^2^; AUC, area under curve.

### Clinicopathological Features

A total of 962 female cases with BC recorded in TCGA were extracted for analyses in this study. The median patient age was 58 years (range, 26–90 years), while the median DSS was 825 days. The 5-years DSS rate for all cases analyzed was 89.4%. BC tumor size, lymph node status, and metastasis status [tumor, node, metastasis (TNM) stage] was defined as outlined by the Eighth Edition American Joint Committee on Cancer (AJCC) Staging Manual (Amin et al., 2017), and molecular subtype (PAM50) was derived from a previous report by Thorsson et al. (2018). The proportion of HER2 subgroup patients in the low-risk group was significantly lower than that in the high-risk group (total dataset, χ^2^ = 92.295, *P* < 0.0001; validation dataset, χ^2^ = 45.400, *P* < 0.0001). Further, a larger tumor size was associated with a higher proportion of patients in the high-risk group for the total dataset (χ^2^ = 18.447, *P* < 0.0001), but not in the validation dataset (χ^2^ = 2.484, *P* = 0.478) (Table 4).

**TABLE 4:** Demographic, clinical, and pathologic characteristics of the patient with breast cancer.

Variable	All dataset					Validation dataset				
	Total	Risk group		χ^2^	*P*-value	Total	Risk group		χ^2^	*P*-value
		Lower	Higher				Lower	Higher		
										
	*n* = 962	*n* = 624	*n* = 338			*n* = 481	321	160		
**Age (years)**										
≤40	87	59	28	0.545	0.762	44	31	13	0.322	0.851
41–60	446	285	161			228	152	76		
≥61	429	280	149			209	138	71		
**Subtype (PAM50)**										
LumA	456	353	103	92.295	0.000	240	186	54	45.400	0.000
LumB	164	90	74			70	41	29		
HER2	67	19	48			33	11	22		
Basal	157	80	77			85	42	43		
Normal	118	82	36			53	41	12		
**Tumor size**										
T1	248	183	65	18.447	0.000	136	98	38	2.484	0.478
T2	560	340	220			274	178	96		
T3	126	88	38			60	38	22		
T4	28	13	15			11	7	4		
**Lymph node status**										
N0	466	299	167	2.551	0.466	253	165	88	1.756	0.624
N1	324	207	117			154	102	52		
N2	107	70	37			45	32	13		
N3	65	48	17			29	22	7		
**Metastasis status**										
M0	948	616	332	0.372	0.542	472	315	157	0.000	0.996
M1	14	8	6			9	6	3		

### A Five-MiRNA Signature Associated With DSS of Patients With BC

**FIGURE 3:**
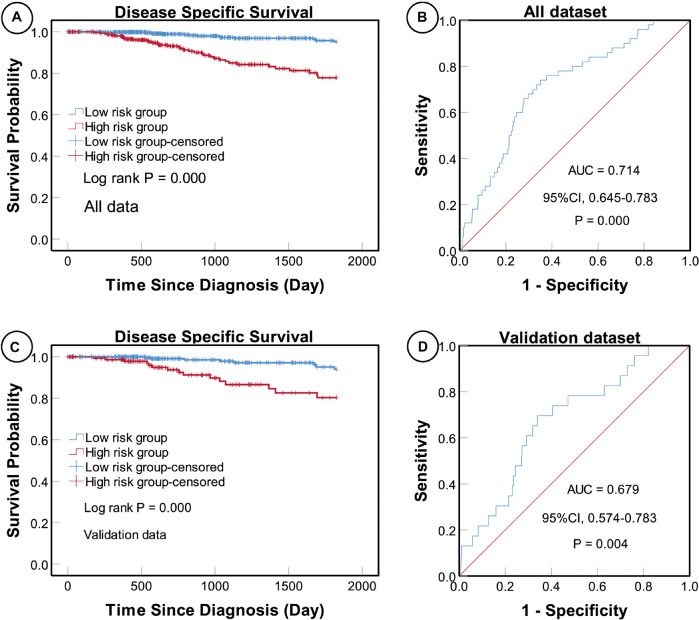
Kaplan–Meier (KM) and receiver operating characteristic (ROC) curves of disease-specific survival (DSS) according to the five-miRNA signature. KM curves of DSS for high- and low-risk groups in the total **(A)** and validation **(C)** datasets. The sensitivity and specificity of the five-miRNA signature for predicting DSS of patients in the total **(B)** and validation **(D)** datasets.

Univariate and multivariate Cox proportional hazard regression analyses indicated that a higher five-miRNA risk score was correlated with higher incidences of clinical events (univariate analysis, HR 5.686, 95% CI: 3.065–10.550, *P* < 0.0001; multivariate analysis, HR 4.376, 95% CI 2.288–8.369, *P* < 0.0001) (Table 5). Moreover, KM survival curves showed that the high-risk group had worse prognosis in both the total (log rank *P* < 0.0001) and validation (log rank *P* < 0.0001) datasets (Figure 3).

**TABLE 5:** Univariate and multivariate Cox proportional hazard models of DSS in breast cancer.

Variables	Univariate			Multivariate		
	HR	95% CI	*P*-value	HR	95% CI	*P*-value
**Age (years)**						
41–60	0.859	0.320–2.302	0.762			
≥61	1.537	0.589–4.014	0.380			
**Subtype (PAM50)**						
LumB	1.228	0.438–3.446	0.696	1.065	0.361–3.140	0.909
HER2	2.481	0.809–7.611	0.112	1.288	0.410–4.046	0.665
Basal	3.981	1.934–8.196	0.000	4.709	2.117–10.475	0.000
Normal	2.658	1.190–5.937	0.017	2.451	1.031–5.827	0.042
**Tumor size**						
T2	1.246	0.590–2.633	0.565	0.701	0.324–1.515	0.366
T3	2.001	0.789–5.071	0.144	1.087	0.401–2.948	0.870
T4	13.460	5.590–32.414	0.000	3.576	1.161–11.020	0.026
**Lymph node status**						
N1	2.332	1.168–4.659	0.016	2.349	1.133–4.871	0.022
N2	3.814	1.630–8.927	0.002	3.245	1.303–8.078	0.011
N3	5.447	2.165–13.704	0.000	3.956	1.145–13.667	0.030
**Metastasis status**						
M1	11.850	5.556–25.280	0.000	3.037	1.184–7.787	0.021
Risk group						
High-risk	5.686	3.065–10.550	0.000	4.376	2.288–8.369	0.000

*DSS, disease-specific survival; CI, confidence interval; HR, hazard ratio.*

### Evaluation of the Predictive Power of the Five-MiRNA Signature

To determine the sensitivity and specificity of the five-miRNA signature for predicting survival, we conducted ROC analyses of the total and validation datasets. The AUC value for the five-miRNA signature was 0.714 (95% CI 0.645–0.783, *P* < 0.0001) in the total dataset and 0.679 (95% CI 0.574–0.783, *P* = 0.004) in the validation dataset, respectively, suggesting that the five-miRNA signature was highly sensitive and specific (Figure 3).

**FIGURE 4:**
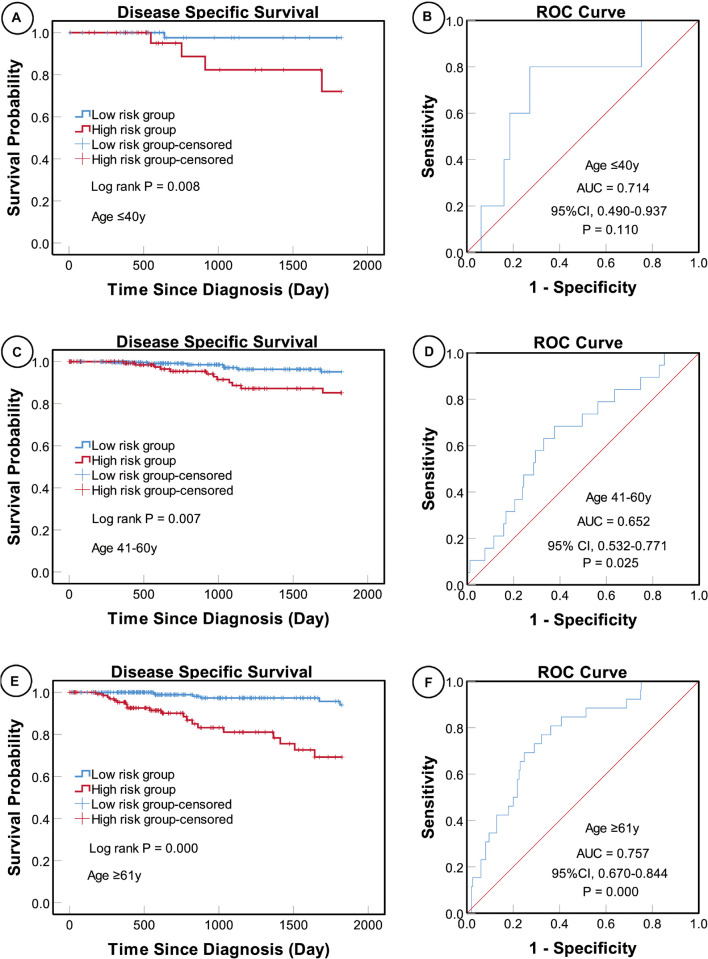
KM and ROC curve analyses of patients stratified by age. KM analysis showed that the patients in low-risk group had significantly better DSS in the ≤ 40 years **(A)**, 41–61 years **(C)**, and ≥ 61 years **(E)** subgroups. ROC analysis showed that the five-miRNA signature exhibited high sensitivity and specificity in predicting the prognosis of patients with BC in the ≤ 40 years **(B)**, 41–61 years **(D)**, and ≥ 61 years **(F)** subgroups.

**FIGURE 5:**
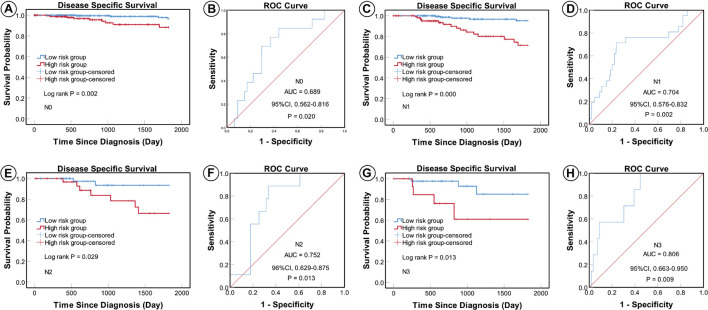
KM and ROC curves for patients stratified by lymph node status. KM analysis showed that patients in the low-risk group had significantly better DSS in the N0 **(A)**, N1 **(C)**, N2 **(E)**, and N3 **(G)** subgroups. ROC analysis showed that the five-miRNA signature exhibited high sensitivity and specificity in predicting the prognosis of patients with BC in the N0 **(B)**, N1 **(D)**, N2 **(F)**, and N3 **(H)** subgroups.

**FIGURE 6:**
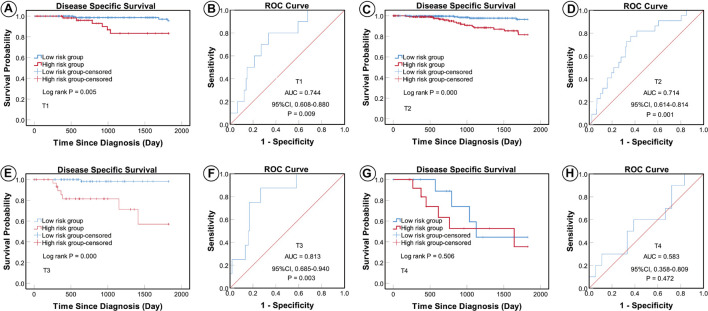
KM and ROC curves for patients stratified by tumor size. KM analysis showed that the patients in the low-risk group had significantly better DSS in the T1 **(A)**, T2 **(C)**, and T3 **(E)** subgroups, but not in the T4 **(G)** subgroup. ROC analysis showed that the five-miRNA signature exhibited high sensitivity and specificity in predicting the prognosis of patients with BC in the T1 **(B)**, T2 **(D)**, and T3 **(F)** subgroups, but not in the T4 **(H)** subgroup.

**FIGURE 7:**
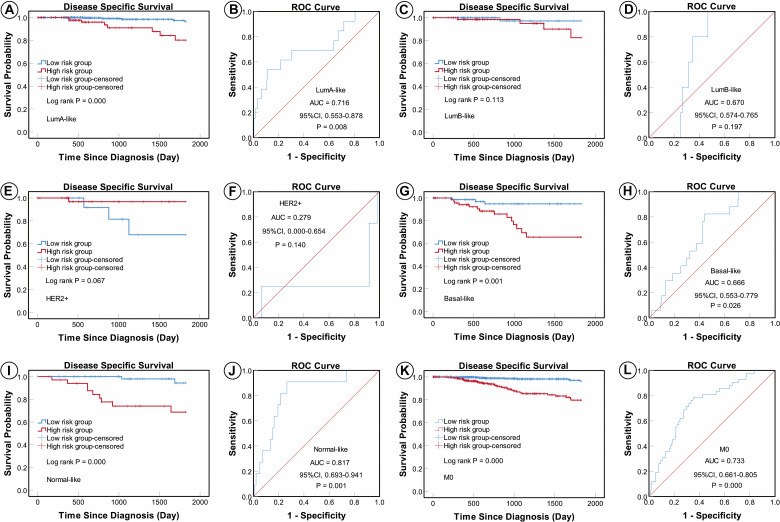
KM and ROC curves for patients stratified by molecular subtype and metastasis status. KM analysis showed that patients in the low-risk group had significantly better DSS in the LumA-like **(A)**, basal-like **(G)**, normal-like **(I)**, and M0 **(K)** subgroups, but not in the LumB-like **(C)** or HER2 **(E)** subgroups. ROC analysis showed that the five-miRNA signature exhibited high sensitivity and specificity in predicting the prognosis of patients with BC in the LumA-like **(B)**, basal-like **(H)**, normal-like **(J)**, and M0 **(L)** subgroups, but not the LumB-like **(D)** or HER2 **(F)** subgroups.

According to the AJCC cancer staging manual (8th edition), TNM stage is correlated with cancer prognosis (Amin et al., 2017). Further, age and intrinsic molecular subtype (PAM50) are also closely linked to prognosis in patients with BC (Anders et al., 2009; Parker et al., 2009; Lænkholm et al., 2018). To validate the potential of the five-miRNA signature as a predictor of DSS of patients with BC, the entire TCGA BC dataset was stratified by cancer stage, age, and molecular subtype; BC cases were split into three age subgroups (≤ 40, 41–60, and ≥ 61 years old), four lymph node status subgroups (N0, N1, N2, and N3), four tumor size subgroups (T1, T2, T3, and T4), and five molecular subtype subgroups (LumA-like, LumB-like, HER2, basal-like, and normal-like). KM analysis indicated that patients in the low-risk group had significantly longer DSS in all three age subgroups (≤ 40 years, *P* = 0.008; 41–61 years *P* = 0.007; and ≥ 61 years, *P* < 0.0001). ROC curve analysis showed that the five-miRNA signature had good sensitivity and specificity for predicting survival in the 41–61 years (AUC = 0.652, 95% CI 0.532–0.771, *P* = 0.025) and the ≥ 61 years (AUC = 0.757, 95% CI 0.670–0.844, *P* < 0.0001) subgroups, but not in the ≤ 40 years (AUC = 0.714, 95% CI 0.490–0.937, *P* = 0.110); KM and ROC curves are presented in Figure 4. In analyses of lymph node, tumor size, molecular subtype, and metastasis status subgroups, KM curves also showed that patients in the low-risk group had significantly better prognosis than those in the high-risk group (N0 subgroup, KM analysis, *P* = 0.002; N1 subgroup, *P* < 0.001; N2 subgroup, *P* = 0.029; N3 subgroup, *P* = 0.013; T1 subgroup, *P* = 0.005; T2 subgroup, *P* < 0.001; T3 subgroup, *P* < 0.001; T4 subgroup, *P* < 0.506; LumA-like subgroup, *P* < 0.001; basal-like subgroup, *P* = 0.001; normal-like subgroup, *P* < 0.001; and M0 subgroup, *P* < 0.001), apart from the LumB-like and HER2 subgroups (LumB-like subgroup, *P* = 0.113; HER2 **+** subgroup, *P* = 0.067). ROC analysis demonstrated that the signature had good sensitivity and specificity for predicting DSS (N0 subgroup, AUC = 0.686, 95% CI 0.562–0.816, *P* = 0.020; N1 subgroup, AUC = 0.704, 95% CI 0.576–0.832, *P* = 0.002; N2 subgroup, AUC = 0.752, 95% CI 0.629–0.875, *P* = 0.013; N3 subgroup, AUC = 0.806, 95% CI 0.663–0.950, *P* = 0.009; T1 subgroup, AUC = 0.744, 95% CI 0.608–0.880, *P* = 0.009; T2 subgroup, AUC = 0.714, 95% CI 0.614–0.814, *P* = 0.001; T3 subgroup, AUC = 0.813, 95% CI 0.685–0.940, *P* = 0.003; LumA-like subgroup, AUC = 0.716, 95% CI 0.553–0.878, *P* = 0.008; basal-like subgroup, AUC = 0.666, 95% CI 0.553–0.779, *P* = 0.026; normal-like subgroup, AUC = 0.817, 95% CI 0.693–0.941, *P* = 0.001; and M0 subgroup, AUC = 0.733, 95% CI 0.661–0.805, *P* < 0.001), but not in the T4, LumB-like, and HER2 subgroups (T4 subgroup, AUC = 0.583, 95% CI 0.358–0.809, *P* = 0.472; LumB-like subgroup, AUC = 0.670, 95% CI 0.574–0.765, *P* = 0.197; HER2 **+** subgroup, AUC = 0.279, 95% CI 0.000–0.654, *P* = 0.140); KM and ROC curves are presented in Figures 5–7, and the results are summarized in Table 6. Overall, these analyses indicate that the five-miRNA signature has a good predictive value.

**TABLE 6:** Result of Kaplan–Meier and ROC analysis based on different regrouping methods.

Regrouping factors	Group	Sample size	Kaplan-Meier	ROC		
			*P*-value	AUC	95% CI	*P*-value
**Age (years)**						
	≤40	87	0.008	0.714	0.490–0.937	0.110
	41–60	446	0.007	0.652	0.532–0.771	0.025
	≥61	429	0.000	0.757	0.670–0.844	0.000
**Subtype (PAM50)**						
	LumA	456	0.000	0.716	0.553–0.878	0.008
	LumB	164	0.113	0.67	0.574–0.765	0.197
	HER2	67	0.067	0.279	0.000–0.654	0.140
	Basal	157	0.001	0.666	0.553–0.779	0.026
	Normal	118	0.000	0.817	0.693–0.941	0.001
**Tumor size**						
	T1	248	0.005	0.744	0.608–0.880	0.009
	T2	560	0.000	0.714	0.614–0.814	0.001
	T3	126	0.000	0.813	0.685–0.940	0.003
	T4	28	0.506	0.583	0.358–0.809	0.472
**Lymph node status**						
	N0	466	0.002	0.689	0.562–0.816	0.020
	N1	324	0.000	0.704	0.576–0.832	0.002
	N2	107	0.029	0.752	0.629–0.875	0.013
	N3	65	0.013	0.806	0.663–0.950	0.009
**Metastasis status**						
	M0	948	0.000	0.733	0.661–0.805	0.000

### Relevance of the MiRNA Signature in Clinical Decision-Making

**FIGURE 8:**
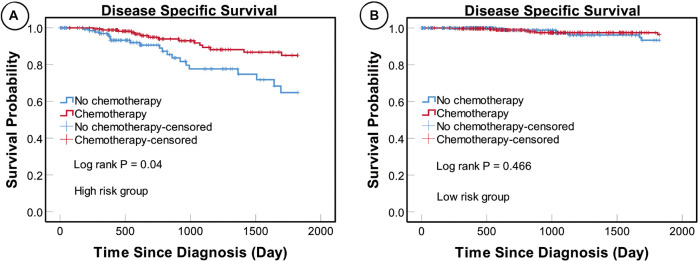
KM curves for patients in different risk subgroups. KM analysis showing that patients in the high-risk **(A)** but not the low-risk **(B)** subgroup who underwent adjuvant chemotherapy had significantly better prognosis than those who did not receive adjuvant chemotherapy.

We found that patients in the high-risk group who underwent adjuvant chemotherapy had significantly better prognosis than those who did not (*P* = 0.004), while no such difference was detected in the low-risk group (*P* = 0.466) (Figure 8). These results suggest that patients in the high-risk group could benefit from adjuvant chemotherapy, while those in the low-risk group may not.

### Nomogram Development

**FIGURE 9:**
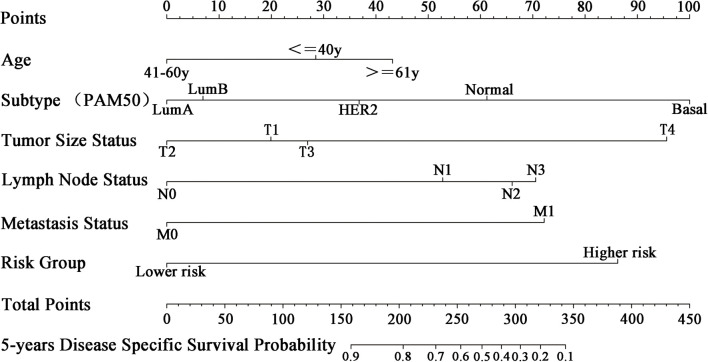
Nomogram for predicting 5-year DSS of patients with BC. Each clinical predictive factor corresponds to a designated point shown by a line drawn perpendicular to the point’s axis. The corresponding sum of risk factor points, shown as total points, can be used to estimate the probability of 5-year DSS by drawing a line straight down to the 5-year DSS axis.

To apply the five-miRNA signature in clinical settings, we combined it with conventional clinical predictors of prognosis (age, tumor size, lymph node, metastasis, and molecular subtype) to create a nomogram (Figure 9). Each risk factor corresponds to a designated point determined by drawing a line perpendicular to the points axis. The corresponding sum of risk factor points located on the total points represents the probability of 5-year DSS by reading straight down to the 5-year DSS axis.

## Discussion

Although, numerous molecular predictors of prognosis have been developed to guide clinical decisions in the management of patients with BC, the scope for application of predictors has been somewhat limited; for example, Oncotype Dx was developed for prediction of prognosis only in cases with ER-positive, HER2-negative, node-negative, and early stage BC (Paik et al., 2004). Numerous studies have demonstrated that miRNAs have potentially important functions in breast tumorigenesis, and multiple miRNAs have been identified as candidate biomarkers for prediction of prognosis in patients with BC (Yan et al., 2008; Chen et al., 2012; Cheng et al., 2012; Zhou et al., 2014). The results of multigene analyses demonstrate that combinations of multiple biomarkers can achieve higher sensitivity and specificity, relative to single-gene biomarkers (Paik et al., 2004; Cardoso et al., 2016).

In the present study, a five-miRNA signature was identified as significantly associated with DSS. Further analyses demonstrated the utility of this five-miRNA signature as a powerful predictor of prognosis in patients with BC. A nomogram constructed by combining the five-miRNA signature and conventional prognostic factors had high value for prediction of 5-year DSS of patients with BC. In the high-risk group, patients who underwent adjuvant chemotherapy had better prognosis than those who did not, but this was not the case in the low-risk group, indicating that patients in the high-risk group could benefit from adjuvant chemotherapy more than those in the low-risk group.

The biological functions of the five miRNAs have been elucidated in numerous experiments, and they are associated with BC prognosis or pathological features. Hsa-miR-30b has been validated as a tumor suppressor that inhibits bone metastasis in BC (Croset et al., 2018). Up-regulation of hsa-miR-210 promotes BC stem cell metastasis, proliferation, and self-renewal by targeting E-cadherin (Tang et al., 2018). Further, hsa-miR-224 promotes tumorigenesis through down-regulation of caspase-9 in triple-negative BC (Zhang et al., 2019), hsa-miR-574 enhances doxorubicin resistance by down-regulating *SMAD4* in BC cells (Sun et al., 2018), and hsa-miR-130a suppresses BC cell migration and invasion (Chen et al., 2018; Kong et al., 2018) and reduces drug resistance in BC (Huang et al., 2019).

Recent research shows that multiple-miRNA signature (model) could predict the prognosis of some cancer, for instance, four-miRNA classifier for esophageal squamous cell carcinoma (Wen et al., 2021), a six-miRNA-based classifier for stage II colon cancer (Zhang et al., 2013), a robust six-miRNA prognostic signature (Zhao and Cui, 2020), and a novel seven-miRNA prognostic model (Lu et al., 2019) for head and neck squamous cell carcinoma. In breast cancer, three multi-miRNA prognostic models have been developed to date (Lai et al., 2019; Tang et al., 2019; Lu et al., 2020). However, our model has several advantages as a predictor of prognosis in patients with BC. First, our study included 962 female cases with BC, while excluding males and cases with missing clinical information, which avoided the possibility of sex-specific effects and ensured more credible results. Second, DSS was chosen as a clinical outcome, rather than OS, as OS is less sensitive for BC-specific mortality. Third, we identified two internal reference miRNAs (hsa-miR-186 and hsa-miR-361), which were essential to normalize expression data for other miRNAs. Quantification methods for miRNA expression levels include relative and absolute expression quantification. Although absolute quantification could detect exact miRNA expression, small changes in an experiment may cause huge relative quantification differences. The purpose of relative quantification is the degree of change in the expression level of the target miRNA relative to the expression level of the reference miRNA, and its function is to correct aspects of operation differences to ensure the accuracy of the experimental results. Also, hsa-miR-186 and hsa-miR-361 were screened by following strict criteria that have been reported by Zhan et al. (2014), they had certain and stable expression levels in tumor and normal tissue, and there was no statistical difference between tumor and normal tissue. So, relative quantification appears to be more robust than absolute quantification approaches. Fourth, patients with high-risk scores using our model were shown to benefit from adjuvant chemotherapy, which could inform clinical decision-making regarding appropriate treatment strategies for patients with BC.

Notwithstanding these strengths, the study had a number of shortcomings and limitations, which should be acknowledged. First, a real-world validation dataset was lacking. Our breast center has already started to establish a validation dataset of BC to verify the findings in this research. However, limited by the follow-up time and the number of cases, it may take a long time. If the validation dataset is consistent with the results of this study, the finding will be applied to a prospective clinical study. Second, the biological functions of the five miRNAs remain to be fully elucidated. Third, KM curve or ROC subgroup analyses did not reveal any significant difference in the subgroups: T4 (*N* = 28), LumB-like (*N* = 164), HER2 (*N* = 67), age ≤ 40 years (*N* = 87), and M1 (*N* = 14); therefore, further study to identify more accurate molecular models for these patient subgroups is warranted. Fourth, while our analyses show that patients with high-risk scores could benefit from adjuvant chemotherapy, which indicates that the five-miRNA signature has theoretical reference significance for individualized clinical decision-making, more clinical studies are necessary to confirm this observation. Fifth, our study lacks an independent validation dataset. In the early stage of study design, we had considered that the full dataset be randomly divided into training and validation datasets by the proportions of 1:1, 3:2, 7:3, and 4:1, but the larger the sample size of the dataset, the higher the credibility of the model established, absolutely selecting full dataset for training and modeling. Although the verification dataset was randomly selected from the total dataset with the overlap of the sample points, it can also verify the reliability of the model.

## Conclusion

In conclusion, we identified a novel five-miRNA prognostic model significantly associated with DSS in patients with BC and developed a nomogram based on the five-miRNA signature with high prognostic prediction value. Moreover, our analyses indicated that patients with high-risk scores using our model could benefit from adjuvant chemotherapy, indicating that the five-miRNA signature has theoretical reference significance for individualized clinical decision-making.

## Data Availability Statement

All datasets generated and analyzed in this study are available from the corresponding author on reasonable request. TCGA datasets analyzed in this study are available at https://cancergenome.nih.gov/ (TCGA repository).

## Ethics Statement

Ethical review and approval was not required for the study on human participants in accordance with the local legislation and institutional requirements. Written informed consent for participation was not required for this study in accordance with the national legislation and the institutional requirements.

## Author Contributions

BT and MH collected, analyzed, interpreted TCGA data, and participated in writing the manuscript. KZ critically revised the manuscript for intellectual content. XQ, YD, and YG controlled the quality of tables and figures. JW and XY designed this study and reviewed the manuscript. All authors contributed to the article and approved the submitted version.

## Conflict of Interest

The authors declare that the research was conducted in the absence of any commercial or financial relationships that could be construed as a potential conflict of interest.

## Publisher’s Note

All claims expressed in this article are solely those of the authors and do not necessarily represent those of their affiliated organizations, or those of the publisher, the editors and the reviewers. Any product that may be evaluated in this article, or claim that may be made by its manufacturer, is not guaranteed or endorsed by the publisher.

## Footnotes

1
https://cancergenome.nih.gov/

## Abbreviations

AJCC: American Joint Committee on Cancer
AUC: area under the curve
BC: breast cancer
CI: confidence intervals
DOC: distance on curve
DSS: disease-specific survival
GDC: Genomic Data Commons
HER2: human epidermal growth factor receptor 2
HR: hazard ratio
KM: Kaplan–Meier
miRNAs: microRNAs
OS: overall survival
ROC: receiver operating characteristic
TCGA: The Cancer Genome Atlas
TNM: tumor size, lymph node status, and metastasis status.
